# Sex- and Age-Specific Optimal Anthropometric Indices as Screening Tools for Metabolic Syndrome in Chinese Adults

**DOI:** 10.1155/2018/1067603

**Published:** 2018-09-17

**Authors:** Jia Zhang, Wenhua Zhu, Lifeng Qiu, Lijuan Huang, Lizheng Fang

**Affiliations:** Department of General Practice, Sir Run Run Shaw Hospital, School of Medicine, Zhejiang University, Hangzhou, China

## Abstract

**Objectives:**

To compare the predictive ability of six anthropometric indices for identification of metabolic syndrome (MetS) and to determine their optimal cut-off points among Chinese adults.

**Methods:**

A total of 59,029 participants were enrolled. Body mass index (BMI), waist circumference (WC), waist-to-height ratio (WHtR), a body shape index (ABSI), body roundness index (BRI), and conicity index (CI) were measured. Receiver-operating characteristic curves analyses were performed to determine the discriminatory power of these indices for the identification of cardiometabolic risks and diagnosis of MetS. The differences in the area under the curve (AUC) values among the indices were evaluated. The Youden index was used to determine the optimal cut-off points.

**Results:**

WHtR and BRI exhibited the highest AUC values for identifying MetS and most cardiometabolic risk factors in both sexes, whereas ABSI showed the lowest AUC value. The general optimal cut-off points in women were 23.03 kg/m^2^ for BMI, 77.25 cm for WC, 0.490 for WHtR, and 3.179 for BRI; those in men were 24.64 kg/m^2^ for BMI, 87.25 cm for WC, 0.510 for WHtR, and 3.547 for BRI. The AUC values and cut-off points of the indices were also analyzed in each age and BMI category.

**Conclusions:**

In Chinese adults, WHtR and BRI showed a superior predictive power for MetS in both sexes, which can be used as simple and effective screening tools for cardiometabolic risks and MetS in clinical practice.

## 1. Introduction

Metabolic syndrome (MetS) is a complex of interrelated metabolic risk factors [1]. According to the National Health and Nutrition Examination Survey (NHANES), more than a third of adults are affected by MetS [2]. Patients with MetS are at a two-fold risk of developing cardiovascular disease (CVD) over the next 5 to 10 years as compared to individuals without the syndrome [1]. Three major risk factors (i.e., high blood pressure, dyslipidemia, and high blood glucose level) explain roughly 50% of cardiovascular outcomes recorded [3].

A growing body of evidence supports that visceral fat plays a role in the development of MetS [4]. The effects of obesity on blood pressure and cholesterol level can account for ~45% of the increased risk of CVD [5]. Hence, it is reasonable that central obesity is defined as a predictor of MetS, and clinical physicians can play a vital role in preventing MetS by identification and management of central obesity.

Use of simple anthropometric indices of body composition, such as body mass index (BMI), waist circumference (WC), and waist-to-height ratio (WHtR), has been considered as a practical and valuable approach to the assessment of adiposity for a long time; however, some studies found that these indices provided limited information on fat distribution. BMI is commonly considered as a valid measure of obesity but may yield a false diagnosis of body fatness [6], especially in young healthy adults [7]. WC is a major clinical parameter used for the indirect evaluation of visceral fat. Nevertheless, it is unclear whether to what extent the range of WC depends on body size [8]. Therefore, more appropriate anthropometric indices that will also take body shape into account and may serve as better indicators of central obesity should be designed.

In 2012, Krakauer and Krakauer proposed a body shape index (ABSI), which was found to be superior to BMI and WC as a measure of metabolic changes and disease risks in the general USA population [9]. A positive association between ABSI and disease risk and mortality hazard was found in some studies [10, 11]. However, other studies obtained opposite results [12, 13]. The body roundness index (BRI), a new index proposed by Thomas et al., is considered a predictor of body fat and visceral adipose tissue volume [14]. Some studies have established the potential of BRI in identifying diabetes and cardiovascular health status [13]; however, limited studies have compared BRI with other anthropometric indices in terms of their ability in predicting cardiometabolic risks. The conicity index (CI), a parameter used to assess body fat distribution [15], has been reported as the most effective indicator of 10-year cardiovascular events [16]. However, a comprehensive consensus has not been reached regarding the best indices for identifying cardiometabolic risks.

Thus, the present study was designed to determine and compare the discriminative performance of the six anthropometric indices as instruments of screening that would best estimate cardiometabolic risks and MetS in Chinese adults. Meanwhile, Chinese populations had the most deleterious abdominal visceral fat distribution and accumulation other than Europeans at a given BMI or WC [17]. Therefore, the present study sought to determine the sex- and age-specific optimal cut-off points for these indices, which could be used as screening tools for cardiometabolic risks in Chinese adults.

## 2. Material and Methods

### 2.1. Study Population

From January 2013 to December 2014, this study consecutively recruited 68,509 participants who underwent routine check-ups at Sir Run Run Shaw Hospital affiliated with Zhejiang University. Participants were eligible if they were aged 18–80 years. All the participants were of Han ethnicity and originated in southeast China. Each participant completed a questionnaire, which included items on medical history and medications under the guidance of physicians, and underwent a physical examination. The exclusion criteria were as follows: (1) pregnancy; (2) medical history of CVDs (e.g., coronary artery disease and myocardial infarction), cerebrovascular diseases, heart failure, arrhythmia, cancer, edema, viral hepatitis, liver cirrhosis, liver and renal impairments, and thyroid dysfunction; (3) treatment with lipid-lowering drugs, corticosteroids, or hormone therapy in the previous 6 months; (4) participation in a weight-loss program or loss of ≥5% of their body weight in the previous 1 year; and (5) consumption of more than 14 ethanol units per week in men and 10 ethanol units in women. Blood sampling, anthropometric measurements, and liver ultrasonography were performed in the morning, after overnight fasting. Finally, 59,029 subjects (22,931 women and 36,098 men) were included in the study.

The study was approved by the Ethics Committee of Sir Run Run Shaw Hospital, Zhejiang University. All participants provided written informed consent before participating in this study.

### 2.2. Anthropometric Indices of Body Composition

Height and weight were measured in light clothing without shoes, using a digital scale. WC was measured using a tape measure placed halfway between the lower border of the ribs and the iliac crest in a horizontal plane at the end of normal expiration. BMI, WHtR, ABSI, BRI, and CI were calculated by the following formulas [9, 14, 16]:

(1) $$\begin{array}{c} BMI=\frac{weight}{{height}^{2}},\\ WHtR=\frac{WC}{height},\\ ABSI=\frac{WC}{\left(BMI^{2/3}\times height^{1/2}\right)},\\ BRI=364.2-365.5\times \sqrt{1-\frac{{\left(WC/2\pi \right)}^{2}}{{\left(0.5\times height\right)}^{2}}},\\ CI=\frac{WC}{0.109\times \sqrt{weight/height}}. \end{array}$$

### 2.3. Clinical and Biochemical Tests

Blood pressure was measured using a standard sphygmomanometer (OMRON 705IT). Two blood pressure recordings were obtained from the right arm of the participants while seated after 10 minutes. The average of the two recordings was used for the statistical analysis. The mean arterial pressure (MAP) was calculated using the following formula: MAP = [systolic blood pressure (SBP) + 2 × diastolic blood pressure (DBP)]/3.

The levels of fasting blood glucose (FBG), serum creatinine (SCr), uric acid (UA), liver enzymes, and lipid panel (including total cholesterol, low-density lipoprotein cholesterol (LDL-C), high-density lipoprotein cholesterol (HDL-C), and triglycerides (TG)) were assessed via standard laboratory procedures using the ARCHITECT c16000 chemistry analyzer.

### 2.4. Definition of Cardiometabolic Risk Factors

High blood pressure was defined as an SBP of ≥140 mmHg or a DBP of ≥90 mmHg. Hypertension was diagnosed if the participants were taking antihypertensive medications or if they had high blood pressure. Dysglycemia included impaired fasting glucose (IFG) and diabetes. IFG was defined as an FBG level of 5.6–7.0 mmol/L, without any history of diabetes or use of antidiabetic medications; diabetes was diagnosed with the presence of a history of diabetes, use of insulin or other antidiabetic medications, or an FBG level of ≥7.0 mmol/L. The participants with one or more of the following results were considered dyslipidemic: high TG level (≥1.69 mmol/L), low HDL-C level (≤1.03 mmol/L for men and ≤1.30 mmol/L for women), and high LDL-C level (≥3.37 mmol/L). Hyperuricemia was diagnosed with the presence of a serum UA level of ≥476 mmol/L in men and ≥393 mmol/L in women.

### 2.5. Diagnosis of Nonalcoholic Fatty Liver Disease (NAFLD)

Liver ultrasonography was performed using the iU22 ultrasound system (Philips Healthcare) with a convex broadband probe (C5-1). The procedure was conducted by an experienced sonographer, who was blinded to the clinical information of the participants.

NAFLD was diagnosed when all of the following conditions were met: (1) hyperechoic texture or bright liver on ultrasound imaging in comparison with the kidney, vascular blurring of the hepatic vein trunk, and deep attenuation in the right hepatic lobe; (2) no significant alcohol consumption; (3) no competing etiologies for hepatic steatosis; and (4) no coexisting causes for chronic liver disease [18].

### 2.6. Definition of MetS and Framingham Point Score

MetS was diagnosed in accordance with the definition of the International Diabetes Federation (IDF) criteria in 2009 and Chinese-specific abdominal obesity standard [1]. The participants were categorized as having MetS when they met three or more of the following components: (1) increased WC (≥90 cm in men; ≥80 cm in women), (2) increased TG level (≥1.7 mmol/L) or drug treatment for such, (3) reduced HDL-C level (<1.03 mmol/L for men; <1.30 mmol/L for women) or drug treatment for such, (4) increased blood pressure (SBP of ≥130 mmHg and/or DBP of ≥85 mmHg) or antihypertensive drug treatment, and (5) increased FBG level (≥5.56 mmol/L) or drug treatment for such. Given that the objective of this study was to establish the optimal measurements for the evaluation of MetS, the requirement of WC for this definition was omitted.

The probability of coronary heart disease (CHD) in 10 years was estimated using the Framingham point score, which was calculated from the NCEP-ATP III algorithm [19].

### 2.7. Statistical Analysis

Statistical analysis was performed using the SPSS V.21.0 (IBM). Data were presented as means and standard deviations for continuous variables and as frequencies and percentages for categorical variables. The descriptive characteristics of the study subjects according to sex-specific blood pressure and blood glucose level were determined using the independent samples *t*-test, ANOVA, nonparametric test (continuous variables), or *χ* ^2^ test (categorical variables). Pearson and partial correlation analyses were applied to calculate the intra- and interobserver variability coefficients. Z-scores for theses anthropometric indices were used in the correlation analyses. Stepwise logistic regression analyses were used to assess the association of the anthropometric indices with each cardiometabolic risk factor, MetS, and Framingham point score. As the presence of diabetes and antidiabetic treatment can influence the subjects' body weight and body composition, the correlation analyses between the anthropometric indices and metabolic components were conducted in the subjects with and without diabetes. The blood pressure, blood glucose level, and corresponding treatment can also affect the subjects' metabolic components; thus, the demographic and clinical characteristics were analyzed in four separate groups: (1) nonhypertensive and nondiabetic, (2) hypertensive, (3) diabetic, and (4) hypertensive and diabetic groups.

Receiver-operating characteristic (ROC) curves and areas under the curve (AUCs) were employed to evaluate the predictive ability of the six anthropometric indices for the identification of cardiometabolic risks and MetS. The optimal cut-off points of the anthropometric indices were also determined according to the values of the indices that maximized the Youden index (sensitivity + specificity – 1). The significance of the difference between two AUC values was assessed using the Hanley and McNeil approach [20]. Differences were considered to be statistically significant at a *p* value of < 0.05.

## 3. Results

Table 1 shows the demographic and clinical characteristics of the 59,029 subjects (22,931 women and 36,098 men) according to the blood pressure and glucose level. 17.2% of the subjects were diagnosed with hypertension; 5.4%, diabetes; 13.3%, IFG; and 28.3% (15.9% women and 36.2% men), MetS. The men had a significantly higher prevalence of hypertension, diabetes, IFG, NAFLD, and MetS; higher BMI, WC, WHtR, ABSI, BRI, CI, FBG, TG, LDL-C, UA, SCr, SBP, and DBP; and lower HDL-C than women. In both sex categories, the subjects with diabetes exhibited significantly higher ABSI, CI, and TG and lower HDL-C than the subjects with hypertension and those without diabetes or hypertension. The subjects with hypertension had significantly higher UA and SCr levels and Framingham point score than the subjects with diabetes and those without hypertension or diabetes.

Table 2 shows the partial correlation between the anthropometric indices and the metabolic components as well as the Framingham point score in the subjects with and without diabetes who were not on antihypertensive or antidiabetic medications. After controlling for age, BMI showed a relatively higher linear correlation with the Framingham point score than the other indices in the subjects with and without diabetes; ABSI showed the weakest correlation.

The results of the logistic regression analyses are shown in Table 3. The odds ratios and 95% confidence intervals were analyzed using 1-standard deviation alteration of the anthropometric indices for the purpose of a more clinically and population-related interpretation. After adjusting for age, WHtR exhibited the highest odds ratios for most cardiometabolic risk factors (diabetes/IFG, high TG, low HDL-C, and hyperuricemia in women and high TG, low HDL-C, and high LDL-C in men) and MetS in both sexes; conversely, ABSI showed the lowest odds ratio.

The AUC values for predicting cardiometabolic risks by each index are shown in Table 4. In both sex categories, WHtR and BRI exhibited the highest AUC values for discriminating most cardiometabolic risk factors (hypertension, diabetes/IFG, high TG, and high LDL-C); BMI showed the highest AUC value for predicting low HDL-C and NAFLD in women and low HDL-C, hyperuricemia, and NAFLD in men. In both sex categories, ABSI exhibited the lowest AUC value for identifying each cardiometabolic risk.

The AUC values for identifying MetS by these indices according to age group are shown in Table 5. Generally, WHtR and BRI exhibited the highest AUC values in both sex categories. In women, BMI showed the highest AUC value in the young population, while WHtR and BRI showed the highest AUC values in both the middle-aged and elderly populations. In men, WHtR and BRI showed the highest AUC values in the young population, while BMI and WC had the highest AUC values in the middle-aged and elderly populations, respectively. It is worth noting that there was no significant difference among the AUC values of BMI, WC, WHtR, and BRI in each age group for men. In both sex categories, ABSI exhibited the lowest AUC value for identifying MetS in each age group.

According to the ROC curve, the cut-off point of BMI that maximized the sensitivity and specificity for predicting the presence of MetS was 23.03 kg/m^2^ in women and 24.64 kg/m^2^ in men; that of WC was 77.25 cm in women and 87.25 cm in men. Alternatively, given that the criteria of the anthropometric indices were used for the first screening for MetS, it is justified to set a cut-off point to obtain a higher sensitivity of at least 80%. With this consideration, the cut-off point for BMI was 22.27 kg/m^2^ in women and 23.91 kg/m^2^ in men, and that of WC was 75.75 cm in women and 84.75 cm in men.

Furthermore, the older population had the larger cut-off points for most indices (WC, WHtR, ABSI, BRI, and CI) in both sex categories, except for BMI. In women, the cut-off points for WC in the young, middle-aged, and elderly populations were 74.75 cm, 78.75 cm, and 80.25 cm, respectively; in men, the cut-off points were 85.80 cm, 87.25 cm, and 88.25 cm, respectively.

The AUC values for identifying MetS by these indices according to BMI category are shown in Table 6. In women, ABSI showed the highest AUC value in the underweight group, whereas WHtR and BRI showed the highest AUC values in the normal weight, overweight, and obesity groups. In men, WHtR and BRI showed the highest AUC values in all BMI categories. Furthermore, a higher cut-off point was observed in the higher BMI category for each anthropometric index than that in the lower BMI categories; the normal BMI category showed the highest AUC value for each index. In women, the cut-off points for WC in the underweight, normal weight, overweight, and obesity groups were 65.25 cm, 74.25 cm, 83.75 cm, and 91.75 cm, respectively; in men, the cut-off points were 73.75 cm, 81.75 cm, 89.90 cm, and 100.25 cm, respectively.

Within each of the four BMI categories, the men and women were further subdivided into groups with higher and lower anthropometric indices according to the cut-off points. Thereafter, logistic regression analyses to determine the association of the cardiometabolic risk factors and MetS with each anthropometric index were conducted in each BMI category; the odds ratios are shown in Table 7. After adjusting for age, the subjects in the groups with higher WC, WHtR, ABSI, BRI, and CI were more likely to present deteriorated metabolic risks and MetS than the groups with lower anthropometric indices. Particularly in the normal weight group, the subjects with higher WC, WHtR, and BRI were approximately two times more likely to have MetS than those with lower indices.

## 4. Discussion

To date, limited studies have compared the ability of ABSI, BRI, and CI in predicting cardiometabolic risks and MetS among Chinese adults with that of classical anthropometric indices, and the optimal cut-off points of these indices were inconclusive. The present study showed that WHtR and BRI can be more effective than either WC or BMI as a marker of MetS and most cardiometabolic risk factors (hypertension, dysglycemia, high TG level, and high LDL-C level) in Chinese population. In addition, the specific cut-off points of the anthropometric indices in each age and BMI category were also determined.

In the present study, patients with a history of CVDs or cerebrovascular diseases were excluded because they are usually treated with multiple medications, such as statins and antidiabetic and antihypertensive medications, which can alter the four components of MetS and influence its diagnosis. Furthermore, these medications have an impact on body weight and body composition, which can cause misunderstanding of the study results. The interaction of these medications should be considered as well. Moreover, these patients are often disabled or experience restricted mobility, which can also influence their body composition. Therefore, patients with CVDs or cerebrovascular diseases were excluded. Patients treated with lipid-lowering agents were also excluded because these medications can affect body weight, BMI, and lipid panel [21]. In addition, statins were mostly used in patients with CVDs or cerebrovascular diseases, who have been excluded from this study. Considering the inclusion and exclusion criteria of this study, the incidence of hypertension, diabetes, dyslipidemia, and MetS might be underestimated. To minimize the impact of metabolic status and medications on the predictive ability of the anthropometric indices and the relationship between such indices and MetS, analyses were conducted in the corresponding subgroups.

### 4.1. BMI

The present study found that BMI was a superior predictor for low HDL-C levels and NAFLD in both sex, but not for the other risk factors or MetS. BMI has long been considered as one of the most commonly used indices; however, its use has limitations. It does not distinguish between muscle mass and adipose tissue nor reflect body fat distribution accurately [22].

Asians have greater total body and abdominal fat distributions and a higher incidence of CVD; they also have more metabolic risk factors than Europeans with the same BMI or WC [23]. A meta-analysis of 239 prospective studies reported that the mortality in CHD had its nadir at 18.5–20.0 kg/m^2^ in East Asia, which is lower than those in other regions [24]. A BMI of 24 kg/m^2^ was recommended as the cut-off point for overweight, with the best sensitivity and specificity for identification of risk factors [25]. In our study, the cut-off point for BMI that maximized the sensitivity and specificity for predicting the presence of MetS was 23.03 kg/m^2^ in women and 24.64 kg/m^2^ in men; the cut-off point decreased to 22.27 kg/m^2^ in women and 23.91 kg/m^2^ in men given a higher sensitivity of at least 80%, which is lower than the WHO-defined normal range.

### 4.2. WC

As one of the essential diagnostic components of MetS, WC has been used as a surrogate marker for abdominal obesity [26]; a number of studies have found that WC predicted mortality risks better than BMI [27, 28]. A recent WHO report summarized evidence for WC as an indicator of disease risks, suggesting that it could be used as an alternative to BMI [29]. The present study showed that WC had a superior predictive ability for MetS compared with BMI but an inferior ability compared with WHtR and BRI. The application of WC has several disadvantages. First, it does not help in distinguishing between visceral and subcutaneous fats [30]; consequently, the other indices based on WC, such as WHtR, ABSI, BRI, and CI, might have this disadvantage. Second, WC does not take height into account; thus, its application may underestimate the relative amount of visceral fat in short populations and overestimate it in tall populations. Thus, without the consideration of height, WC might be misleading when diagnosing central obesity and consequently cardiometabolic risks.

Notably, the recommended WC threshold for abdominal obesity in Asian adults is ≥90 cm in men and ≥80 cm in women according to the IDF [1] and the expert consultation for the WHO [31]. In China, the cut-off points of ≥85 cm in men and ≥80 cm in women have been suggested [25]. In the present study, the general optimal cut-off point of WC for identifying MetS was 77.25 cm in women and 87.25 cm in men, and that in terms of a high sensitivity was 75.75 cm in women and 84.75 cm in men. The cut-off points of WC described in this study is smaller than those proposed by the WHO and IDF [32], and the decreased WC cut-off points could be applied in clinical practice to screen for MetS in Chinese adults.

In addition, the higher cut-off points of WC were observed in the higher BMI categories in the present study. The optimal cut-off points in the underweight, normal weight, overweight, and obesity groups in women were 65.25 cm, 74.25 cm, 83.75 cm, and 91.75 cm, respectively; those in men were 73.75 cm, 81.75 cm, 89.90 cm, and 100.25 cm, respectively. Considering these results, lean individuals seem to have a more rigorous threshold for WC than heavier individuals. This result is consistent with those of another study [33], which found that a high WC phenotype was associated with a more detrimental cardiometabolic risk profile and that a substantial variability in WC remains at any given BMI. Such results also emphasize the limitations of using BMI or WC in isolation as adiposity indices as they do not individually reflect the heterogeneity of abdominal adiposity (and/or muscle mass) and related cardiometabolic risks. Indeed, their use in isolation may lead to an underestimation of abdominal obesity-related risks in a substantial proportion of patients [33]. Hence, BMI should be taken into consideration when using WC to diagnose central obesity, and the combined application of BMI and WC might be a more effective and justified method when screening for central obesity and MetS at a given BMI.

### 4.3. WHtR

In our study, WHtR was shown as a superior predictor for hypertension, dysglycemia, high TG level, high LDL-C level, and MetS compared with BMI, WC, ABSI, and CI in both sexes. This finding is in accord with those of previous studies, which showed that WHtR can predict MetS superiorly to BMI and WC [34–36]. The mechanism of the superiority of WHtR in predicting MetS might be that its application takes height into consideration, which will better reflect central obesity. Therefore, WHtR might be a favorable screening tool for cardiometabolic risks and MetS in Chinese adults; however, more studies are required to determine the optimal cut-off point of WHtR.

### 4.4. BRI

BRI is a newly developed index based on three separate databases: NHANES III, studies conducted at St. Luke's/Roosevelt Hospital New York Nutrition Obesity Research Center, and studies conducted at Christian Albrecht's University in Kiel, Germany. The race groups included Whites, Hispanics, African Americans, Mexican Americans, and “others” (Aleuts, Eskimos, American Indians, Asians, or Pacific Islanders). BRI has been found as a better predictor of both body fat and visceral adipose tissue volume than BMI and WC [14]; however, limited studies have compared BRI with the other anthropometric indices in terms of their ability to predict MetS and CVD risks. Some studies have found a potential for BRI to identify diabetes and cardiometabolic risks [13, 37, 38], whereas other studies have shown that BRI was not superior to the classical indices in predicting MetS [39, 40]. Our study was the first to determine the ability of BRI to identify each cardiometabolic risk factor and MetS according to age, sex, and BMI categories. In the present study, BRI showed a superior predictive ability to identify hypertension, dysglycemia, high TG level, high LDL-C level, and MetS compared with BMI, WC, ABSI, and CI.

It is worth noting that WHtR exhibited the highest odds ratio for MetS, and WHtR and BMI showed the highest odds ratios for most cardiometabolic risk factors in the logistic regression analyses. However, WHtR and BRI had the highest AUC values for discriminating most cardiometabolic risk factors and MetS. These discrepancies might be explained by the different statistical analyses performed. With odds ratios and 95% confidence intervals, logistic regression analyses were performed to quantify how strongly the anthropometric indices were associated with the metabolic risks. ROC curve analyses were used to assess the accuracy or usefulness of the predictions for the metabolic risks that maximized the sensitivity and specificity for predictions. Although BMI showed the strongest association with some cardiometabolic risks, its disadvantage might result in the inaccuracy when predicting cardiometabolic risks.

### 4.5. ABSI and CI

ABSI was proposed on the basis of data from the NHANES, which was designed to sample a civilian noninstitutionalized USA population using a cluster approach, including Mexicans, Hispanics, Whites, Blacks, and other ethnicities [9]. Several studies have found that ABSI was superior to the BMI and WC as a measure of metabolic changes and that it showed a stronger association with total, cardiovascular, and cancer mortalities in USA, Poland, and Netherlands populations [41–43]. However, some Chinese studies demonstrated that ABSI seemed to be a weaker index for predicting MetS and CVD risks than the BMI and WC [10, 12, 13, 44]. Similar results were obtained in a study conducted on a Japanese population [45]. A study in South Korea proposed a modified ABSI, i.e., z-score of the log-transformed ABSI, which is a new measure of abdominal obesity with the ability to predict hypertension and impaired health-related quality of life, irrespective of the BMI [46]. In the present study, ABSI exhibited the lowest odds ratio for cardiometabolic risk factors, MetS, and Framingham point score and the lowest predictive ability for cardiometabolic risks and MetS; this finding suggests that the ABSI could hardly be used to identify cardiometabolic risks or MetS in Chinese or Asian adults. Further studies should be conducted to modify the ABSI for better application in this population.

CI is a parameter that takes into consideration the abdominal girth, weight, and height; it was found as an equivalent health indicator to the waist-to-hip ratio (WHR) in European and USA populations [47]. It was claimed that CI has several advantages over the WHR: it has a theoretical range; includes a built-in adjustment of WC for height and weight, allowing direct comparisons of abdominal adiposity between individuals or even between populations; and does not require the hip circumference to assess fat distribution. It has also been reported as the most effective indicator of 10-year cardiovascular events among obesity indices [16]. However, the Framingham Heart Study found that CI was not associated with an increase in CHD incidence or mortality and was an inferior marker compared with BMI for predicting CHD incidence and mortality [48]. Our study showed that CI was not a superior predictor for MetS compared with BMI, WC, WHtR, and BRI, which is consistent with those of other studies conducted on Chinese population [49, 50]. These discrepancies might be explained by the varied ethnicity, size, and characteristics of study samples.

### 4.6. Sex and Age Effects

An obvious difference was observed in the cut-off points of each index between two sexes, suggesting that sex-specific references should be used in clinical practice. Besides, the analyses found a stronger correlation between WC and BMI in men than in women (see Supplementary Materials (available here)). This phenomenon could be explained by the difference in the body composition and fat distribution between men and women. For any given WC or BMI, men have a larger amount of visceral adipose tissue, whereas women have a larger amount of subcutaneous adipose tissue [51].

In addition, the results indicated that the younger population had a lower threshold for the central obesity index than the older population. A meta-analysis also found similar results, which showed that the hazard rates for overweight and obesity were higher at younger ages than older ages; further, the nadir depended on age, with a BMI of 22 kg/m^2^ for baseline ages 35–49 years, 23 kg/m^2^ for baseline ages 50–69 years, and 24 kg/m^2^ for baseline ages 70–89 years [24]. Therefore, physicians should pay attention to the age of patients when screening for MetS, and the age-specific threshold could be applied in clinical practice. Besides, the AUC values of the indices (i.e., BMI, WC, WHtR, BRI, and CI) tended to decrease with age in the women, suggesting that the predictive ability of these indices for MetS was weaker in the older women, which is in line with those of another study [52]. This trend might be explained by the fact that the subcutaneous fat, rather than the visceral fat, accumulates with increasing age in women. Since the anthropometric indices for central obesity cannot distinguish visceral fat from subcutaneous fat, the discriminative ability of the indices for MetS may get weaker in older women.

### 4.7. Limitations

The present study has several limitations. First, this study was a cross-sectional study, which cannot establish a cause-effect relationship or provide any mechanistic explanation for the results. Second, all participants in this study were of Chinese ethnicity and residents of southeast China, who were recruited from a single hospital. Third, information on the diet and exercise of the subjects was not obtained in this study; these could be confounding factors in the relationship between anthropometric indices and cardiometabolic risks. Finally, the 2 h postprandial glucose level has not been tested, which may have led to the underdiagnosis of some subjects with diabetes.

A high prevalence of NAFLD was observed in the present study, which may be caused by the unhealthy diet and sedentary lifestyle of the population in southeast China. NAFLD has been recognized as the hepatic manifestation of MetS and insulin resistance, and it is characterized by a remarkable decreased insulin effects on both glucose and lipid metabolism [53]. This hepatic insulin resistance leads to the overproduction of glucose and stimulation of insulin secretion [54]. The fatty liver also overproduces triglyceride-rich very low-density lipoprotein in the fasting state [55, 56] and during hyperinsulinemia [57], leading to hypertriglyceridemia and a low HDL cholesterol concentration [58]. It is a remarkable fact that the association of NAFLD with features of MetS was independent of BMI; NAFLD patients had a relatively increased visceral adiposity even in the presence of normal body weight [53]. In the present study, the high prevalence of NAFLD may be responsible for the high prevalence of MetS, and the presence of NAFLD could impact the analyses of the association between anthropometric indices and MetS. Further studies are needed to analyze the relationship between MetS, obesity indices, and NAFLD.

## 5. Conclusion

In the present study, WHtR and BRI showed superior predictive abilities for MetS in both Chinese men and women. Sex- and age-specific optimal cut-off points were obtained for each anthropometric index in the Chinese adults, which can be easily used in clinical practice. The combined application of BMI and the other central obesity indices might be a more effective and justified method when screening for central obesity and MetS at a given BMI.

## Figures and Tables

**Table 1:** General demographic and clinical characteristics of the study population according to blood pressure and glucose level in the female and male groups.

		Global *n* = 59,029	Nonhypertensive and nondiabetic *n* = 47,019 (79.7)	Only hypertensive *n* = 8,822 (14.9)	Only diabetic *n* = 1,841 (3.1)	Hypertensive and diabetic *n* = 1,347 (2.3)	*p*
Age (years)	F	44.02 ± 10.96	42.25 ± 10.09	54.85 ± 9.48	53.60 ± 9.41	59.90 ± 9.02	<0.001^a^
	M	45.34 ± 10.98	43.40 ± 10.40	50.90 ± 10.85	50.48 ± 8.92	54.75 ± 10.18	<0.001^a^
Sex (%)	F	22,931	19,799 (86.3)	2478 (10.8)	351 (1.5)	303 (1.3)	<0.001^b^
	M	36,098	27,220 (75.4)	6344 (17.6)	1490 (4.1)	1044 (2.9)	

*Anthropometric indices*							
BMI (kg/m^2^)	F	22.64 ± 2.99	22.28 ± 2.79	24.83 ± 3.12	24.72 ± 3.50	25.76 ± 3.49	<0.001^a^
	M	24.89 ± 3.12	24.45 ± 3.02	26.20 ± 2.99	25.88 ± 2.98	27.23 ± 3.24	<0.001^a^
WC (cm)	F	76.28 ± 8.58	75.18 ± 8.02	82.73 ± 8.52	83.55 ± 9.38	86.99 ± 8.75	<0.001^a^
	M	87.71 ± 8.69	86.37 ± 8.41	91.38 ± 8.08	91.36 ± 8.15	95.13 ± 8.73	<0.001^a^
High WC (*n*, %)	F	7608 (33.2)	5545 (28.0)	1589 (64.1)	226 (64.5)	248 (81.8)	<0.001^a^
	M	23,460 (65.0)	16,174 (59.4)	5145 (81.1)	1194 (80.1)	947 (90.7)	<0.001^a^
WHtR	F	0.48 ± 0.06	0.47 ± 0.05	0.53 ± 0.06	0.53 ± 0.06	0.56 ± 0.06	<0.001^a^
	M	0.52 ± 0.05	0.51 ± 0.05	0.54 ± 0.05	0.54 ± 0.05	0.56 ± 0.05	<0.001^a^
ABSI	F	0.0759 ± 0.0047	0.0755 ± 0.0046	0.0778 ± 0.0048	0.0788 ± 0.0045	0.0800 ± 0.0046	<0.001^a^
	M	0.0791 ± 0.0039	0.0787 ± 0.0039	0.0798 ± 0.0037	0.0804 ± 0.0037	0.0810 ± 0.0037	<0.001^a^
BRI	F	3.09 ± 1.07	2.94 ± 0.97	3.97 ± 1.14	4.08 ± 1.26	4.57 ± 1.22	<0.001^a^
	M	3.73 ± 1.01	3.56 ± 0.96	4.19 ± 0.96	4.19 ± 0.99	4.67 ± 1.10	<0.001^a^
CI	F	1.169 ± 0.080	1.161 ± 0.077	1.218 ± 0.079	1.232 ± 0.076	1.260 ± 0.074	<0.001^a^
	M	1.238 ± 0.067	1.229 ± 0.066	1.260 ± 0.061	1.270 ± 0.060	1.288 ± 0.063	<0.001^a^

*Biochemical indices*							
FBG level (mmol/L)	F	5.02 ± 0.81	4.90 ± 0.47	5.17 ± 0.57	8.24 ± 2.51	7.68 ± 2.08	<0.001^a^
	M	5.34 ± 1.24	5.05 ± 0.54	5.28 ± 0.60	8.65 ± 2.69	8.41 ± 2.38	<0.001^a^
TG level (mmol/L)	F	1.22 ± 0.95	1.14 ± 0.85	1.68 ± 1.14	1.99 ± 1.99	2.24 ± 1.77	<0.001^a^
	M	1.94 ± 1.62	1.82 ± 1.44	2.12 ± 1.55	2.70 ± 2.78	2.86 ± 3.02	<0.001^a^
High TG level (*n*, %)	F	3886 (16.9)	2681 (13.5)	893 (36.0)	153 (43.6)	159 (52.5)	<0.001^a^
	M	15,622 (43.3)	10,807 (39.7)	3296 (52.0)	865 (58.1)	654 (62.6)	<0.001^a^
HDL-C level (mmol/L)	F	1.31 ± 0.30	1.32 ± 0.30	1.26 ± 0.29	1.20 ± 0.28	1.15 ± 0.25	<0.001^a^
	M	1.10 ± 0.26	1.11 ± 0.25	1.10 ± 0.26	1.03 ± 0.24	1.06 ± 0.27	<0.001^a^
Low HDL-C level (*n*, %)	F	12,241 (53.4)	10,247 (51.8)	1513 (61.1)	256 (72.9)	225 (74.3)	<0.001^a^
	M	16,501 (45.7)	12,155 (44.7)	2926 (46.1)	852 (57.2)	568 (54.4)	<0.001^a^
LDL-C level (mmol/L)	F	2.88 ± 0.81	2.84 ± 0.79	3.19 ± 0.88	3.18 ± 0.91	3.08 ± 1.01	<0.001^a^
	M	3.06 ± 0.84	3.06 ± 0.83	3.10 ± 0.84	3.01 ± 0.94	2.98 ± 0.96	<0.001^a^
Uric acid level (*μ*mol/L)	F	281.75 ± 61.66	277.44 ± 58.46	308.57 ± 70.35	291.56 ± 72.16	332.59 ± 89.91	<0.001^a^
	M	399.45 ± 79.80	397.12 ± 77.52	417.46 ± 84.23	372.79 ± 80.73	388.64 ± 88.56	<0.001^a^
SCr level (*μ*mol/L)	F	57.63 ± 9.06	57.48 ± 8.73	59.28 ± 10.64	53.64 ± 10.05	57.92 ± 12.00	<0.001^a^
	M	79.90 ± 12.08	80.20 ± 11.35	80.78 ± 13.18	73.39 ± 12.84	76.05 ± 17.57	<0.001^a^
TG/HDL-C ratio	F	1.073 ± 2.653	0.986 ± 2.750	1.506 ± 1.609	1.901 ± 2.514	2.22 ± 2.42	<0.001^a^
	M	1.990 ± 2.536	1.852 ± 2.349	2.137 ± 2.036	3.104 ± 4.746	3.096 ± 4.177	<0.001^a^

*Cardiometabolic risks*							
NAFLD (*n*, %)	F	3918 (17.1)	2571 (13.0)	982 (39.6)	179 (51.0)	186 (61.4)	<0.001^a^
	M	16,164 (44.8)	10,812 (39.7)	3608 (56.9)	983 (66.0)	761 (72.9)	<0.001^a^
Hypertension (*n*, %)	F	2781 (12.1)	—	—	—	—	<0.001^b^
	M	7388 (20.5)	—	—	—	—	
Antihypertensive drugs (*n*, %)	F	1747 (7.6)	—	1522 (61.4)	—	224 (73.9)	<0.001^b^
	M	3897 (10.8)	—	3220 (50.8)	—	675 (64.7)	
SBP (mmHg)	F	117.50 ± 16.50	113.94 ± 13.39	141.40 ± 15.63	126.04 ± 13.94	145.09 ± 17.48	<0.001^a^
	M	126.51 ± 14.62	122.55 ± 11.85	140.97 ± 14.74	125.97 ± 12.28	142.55 ± 16.16	<0.001^a^
DBP (mmHg)	F	70.64 ± 10.72	68.96 ± 9.64	82.46 ± 10.94	74.09 ± 9.93	79.56 ± 10.61	<0.001^a^
	M	77.48 ± 10.74	74.99 ± 9.37	86.96 ± 10.66	77.25 ± 9.13	85.25 ± 11.05	<0.001^a^
Diabetes (*n*, %)	F	654 (2.9)	—	—	—	—	<0.001^b^
	M	2534 (7.0)	—	—	—	—	
IFG (*n*, %)	F	2098 (9.1)	1570 (7.9)	528 (21.3)	—	—	<0.001^b^
	M	5761 (16.0)	4000 (14.7)	1761 (27.8)	—	—	
Antidiabetic drugs (*n*, %)	F	355 (1.5)	—	—	181 (51.6)	174 (57.4)	<0.001^b^
	M	1073 (3.0)	—	—	570 (38.3)	503 (48.2)	
Dyslipidemia (*n*, %)	F	12,988 (56.6)	10,775 (54.4)	1698 (68.5)	269 (76.6)	246 (81.2)	<0.001^a^
	M	22,407 (62.1)	16,201 (59.5)	4289 (67.6)	1109 (74.4)	808 (77.4)	<0.001^a^
Hyperuricemia (*n*, %)	F	1097 (4.8)	693 (3.5)	303 (12.2)	33 (9.4)	68 (22.4)	<0.001^a^
	M	5730 (15.9)	4004 (14.7)	1398 (22.0)	165 (11.1)	163 (15.6)	<0.001^a^
MetS (*n*, %)	F	3654 (15.9)	1647 (8.3)	1506 (60.8)	215 (61.3)	286 (94.4)	<0.001^a^
	M	13,069 (36.2)	6660 (24.5)	4392 (69.2)	1009 (67.7)	1008 (96.6)	<0.001^a^
Framingham point score	F	4.404 ± 2.804	4.026 ± 2.650	6.961 ± 2.480	5.388 ± 2.726	7.259 ± 2.370	<0.001^a^
	M	4.877 ± 2.470	4.754 ± 2.493	5.368 ± 2.339	4.686 ± 2.376	5.405 ± 2.339	<0.001^a^

F: female; M: male; BMI: body mass index; WC: waist circumference; WHtR: waist-to-height ratio; ABSI: a body shape index; BRI: body roundness index; CI: conicity index; FBG: fasting blood glucose; TG: triglycerides; HDL-C: high-density lipoprotein cholesterol; LDL-C: low-density lipoprotein cholesterol; SCr: serum creatinine; NAFLD: nonalcoholic fatty liver disease; SBP: systolic blood pressure; DBP: diastolic blood pressure; IFG: impaired fasting glucose; MetS: metabolic syndrome. Data for qualitative variables are expressed as *n* (%) and quantitative variables as mean ± standard deviation. ^a^ *p* < 0.001, comparison between groups according to blood pressure and glucose level. ^b^ *p* < 0.001, comparison between women and men.

**Table 2:** Partial correlations between the anthropometric indices and metabolic components in the nondiabetic and diabetic groups without medications.

Index	Nondiabetic								Diabetic without medications							
	SBP	DBP	FBG	TG	HDL-C	LDL-C	UA	Framingham point score	SBP	DBP	FBG	TG	HDL-C	LDL-C	UA	Framingham point score
*Female*																
BMI	**0.264 ** ^**a**^	**0.218 ** ^**a**^	**0.190 ** ^**a**^	0.220^a^	−0.261^a^	**0.144 ** ^**a**^	**0.253 ** ^**a**^	**0.265 ** ^**a**^	—	0.198^b^	—	—	−0.197^b^	—	0.350^a^	**0.222 ** ^**a**^
WC	0.235^a^	0.198^a^	0.168^a^	0.222^a^	−0.265^a^	0.130^a^	0.244^a^	0.244^a^	—	0.186^b^	—	—	**−0.237 ** ^**a**^	—	**0.360 ** ^**a**^	0.212^a^
WHtR	0.236^a^	0.199^a^	0.165^a^	**0.229 ** ^**a**^	**−0.267 ** ^**a**^	0.136^a^	0.241^a^	0.249^a^	—	0.177^c^		—	−0.232^b^	—	0.338^a^	0.201^b^
ABSI	0.036^a^	0.039^a^	0.019^a^	0.081^a^	−0.094^a^	0.028^a^	0.063^a^	0.055^a^	—	—	—	—	—	—	0.109^a^	—
BRI	0.241^a^	0.202^a^	0.169^a^	0.229^a^	−0.257^a^	0.134^a^	0.243^a^	0.246^a^	—	0.179^b^	—	—	−0.209^b^	—	0.332^a^	0.197^b^
CI	0.120^a^	0.107^a^	0.080^a^	0.149^a^	0.176^a^	0.074^a^	0.142^a^	0.139^a^	—	—	—	—	−0.213^b^	—	0.241^a^	0.133^c^
*Male*																
BMI	**0.298 ** ^**a**^	**0.295 ** ^**a**^	**0.239 ** ^**a**^	0.267^a^	−0.299^a^	0.153^a^	0.304^a^	**0.338 ** ^**a**^	**0.221 ** ^**a**^	**0.199 ** ^**a**^	−0.077^b^	—	−0.166^a^	—	**0.172 ** ^**a**^	**0.211 ** ^**a**^
WC	0.255^a^	0.268^a^	0.224^a^	0.270^a^	**−0.307 ** ^**a**^	0.144^a^	**0.307 ** ^**a**^	0.326^a^	0.188^a^	0.197^a^	—	0.059^c^	**−0.200 ** ^**a**^	—	**0.172 ** ^**a**^	0.194^a^
WHtR	0.255^a^	0.270^a^	0.219^a^	**0.277 ** ^**a**^	−0.301^a^	**0.155 ** ^**a**^	0.297^a^	0.336^a^	0.185^a^	0.186^a^	—	**0.078 ** ^**b**^	−0.184^a^	—	0.153^a^	0.199^a^
ABSI	—	0.034^a^	0.030^a^	0.092^a^	−0.098^a^	0.038^a^	0.087^a^	0.087^a^	—	—	**0.084 ** ^**b**^	—	−0.105^a^	—	—	—
BRI	0.253^a^	0.268^a^	0.220^a^	0.273^a^	−0.294^a^	0.149^a^	0.293^a^	0.326^a^	0.184^a^	0.182^a^	—	0.075^c^	−0.176^a^	—	0.152^a^	0.192^a^
CI	0.120^a^	0.152^a^	0.127^a^	0.194^a^	−0.214^a^	0.099^a^	0.204^a^	0.219^a^	0.086^a^	0.120^a^	—	0.068^c^	−0.163^a^	—	0.102^a^	0.120^a^

BMI: body mass index; WC: waist circumference; WHtR: waist-to-height ratio; ABSI: a body shape index; BRI: body roundness index; CI: conicity index; SBP: systolic blood pressure; DBP: diastolic blood pressure; FBG: fasting blood glucose; TG: triglycerides; HDL-C: high-density lipoprotein cholesterol; LDL-C: low-density lipoprotein cholesterol; UA: uric acid. Z-scores for theses anthropometric indices were used in the correlation analyses. The *r* of the correlation was adjusted for age. ^a^ *p* < 0.001, ^b^ *p* < 0.01, ^c^ *p* < 0.05. The bold indicates the highest value of *r* among the indices.

**Table 3:** Logistic regression analyses of the association of the cardiometabolic risk factors and MetS with each anthropometric index, illustrated by the ORs and 95% CIs for 1-SD alteration of the anthropometric indices.

Index	Hypertension		Diabetes/IFG		High TG		Low HDL-C		High LDL-C		Hyperuricemia		NAFLD		MetS (two components other than WC)	
	OR	95% CI	OR	95% CI	OR	95% CI	OR	95% CI	OR	95% CI	OR	95% CI	OR	95% CI	OR	95% CI
*Female*																
BMI	**1.930** ^**a**^	1.839–2.026	1.673^a^	1.605–1.743	1.867^a^	1.798–1.939	1.701^a^	1.648–1.755	**1.320** ^**a**^	1.278–1.363	1.901^a^	1.797–2.010	**3.705** ^**a**^	3.532–3.887	2.241^a^	2.157–2.329
WC	1.700^a^	1.622–1.781	1.696^a^	1.622–1.772	1.927^a^	1.850–2.006	1.757^a^	1.700–1.815	1.292^a^	1.248–1.337	1.960^a^	1.843–2.083	3.579^a^	3.407–3.760	2.254^a^	2.165–2.347
WHtR	1.721^a^	1.640–1.807	**1.733** ^**a**^	1.655–1.814	**1.995** ^**a**^	1.913–2.082	**1.803** ^**a**^	1.742–1.866	1.319^a^	1.273–1.367	**2.015** ^**a**^	1.891–2.147	3.613^a^	3.436–3.800	**2.348** ^**a**^	2.251–2.448
ABSI	—	—	1.154^a^	1.104–1.206	1.225^a^	1.178–1.274	1.195^a^	1.161–1.230	1.041^c^	1.007–1.077	1.204^a^	1.129–1.285	1.241^a^	1.195–1.290	1.224^a^	1.180–1.269
BRI	1.646^a^	1.572–1.723	1.676^a^	1.605–1.750	1.891^a^	1.816–1.968	1.779^a^	1.718–1.842	1.292^a^	1.248–1.338	1.889^a^	1.782–2.002	3.353	3.196–3.519	2.250^a^	2.160–2.344
CI	1.279^a^	1.218–1.343	1.402^a^	1.339–1.469	1.550^a^	1.488–1.615	1.434^a^	1.390–1.479	1.153^a^	1.113–1.194	1.559^a^	1.458–1.667	1.937^a^	1.856–2.022	1.638^a^	1.575–1.703
*Male*																
BMI	**1.956** ^**a**^	1.899–2.014	**1.749** ^**a**^	1.703–1.797	2.029^a^	1.980–2.080	1.689^a^	1.651–1.728	1.273^a^	1.245–1.301	1.690^a^	1.642–1.740	**3.181** ^**a**^	3.087–3.277	2.423^a^	2.359–2.489
WC	1.837^a^	1.783–1.892	1.726^a^	1.679–1.774	2.098^a^	2.046–2.151	1.706^a^	1.667–1.747	1.270^a^	1.242–1.299	**1.730** ^**a**^	1.679–1.783	3.117^a^	2.025–3.210	2.399^a^	2.335–2.464
WHtR	1.860^a^	1.805–1.917	1.736^a^	1.689–1.785	**2.158** ^**a**^	2.103–2.214	**1.713** ^**a**^	1.673–1.754	**1.301** ^**a**^	1.271–1.331	1.717^a^	1.666–1.770	3.101^a^	3.010–3.195	**2.455** ^**a**^	2.389–2.523
ABSI	1.073^a^	1.043–1.104	1.123^a^	1.094–1.154	1.288^a^	1.259–1.318	1.169^a^	1.144–1.196	1.086^a^	1.061–1.112	1.170^a^	1.135–1.206	1.268^a^	1.240–1.297	1.238^a^	1.120–1.267
BRI	1.807^a^	1.756–1.860	1.705^a^	1.660–1.752	2.108^a^	2.055–2.163	1.689^a^	1.649–1.729	1.282^a^	1.253–1.312	1.665^a^	1.617–1.714	3.039^a^	2.950–3.130	2.410^a^	2.346–2.477
CI	1.405^a^	1.364–1.446	1.415^a^	1.376–1.455	1.721^a^	1.679–1.764	1.449^a^	1.416–1.483	1.203^a^	1.175–1.232	1.474^a^	1.428–1.521	1.961^a^	1.911–2.011	1.757^a^	1.713–1.801

MetS: metabolic syndrome; OR: odds ratio; 95% CI: 95% confidence interval; SD: standard deviation; BMI: body mass index; WC: waist circumference; WHtR: waist-to-height ratio; ABSI: a body shape index; BRI: body roundness index; CI: conicity index; IFG: impaired fasting glucose; TG: triglycerides; HDL-C: high-density lipoprotein cholesterol; LDL-C: low-density lipoprotein cholesterol; NAFLD: nonalcoholic fatty liver. The regression analyses were adjusted for age. The ORs and 95% CIs were analyzed using 1-SD alteration of the anthropometric indices. ^a^ *p* < 0.001, ^b^ *p* < 0.01, ^c^ *p* < 0.05. The bold indicates the highest value of odds ratio among the indices.

**Table 4:** AUCs, optimal cut-off points, and sensitivities and specificities of the cut-off points of the anthropometric indices for predicting cardiometabolic risk factors in the ROC analyses.

Components	BMI			WC			WHtR			ABSI			BRI			CI		
	AUC (95% CI)	Cut-off	Sen Spe	AUC (95% CI)	Cut-off	Sen Spe	AUC (95% CI)	Cut-off	Sen Spe	AUC (95% CI)	Cut-off	Sen Spe	AUC (95% CI)	Cut-off	Sen Spe	AUC (95% CI)	Cut-off	Sen Spe
*Women*																		
Hypertension	0.738^ac^ (0.728–0.748)	23.18	0.704 0.663	0.752^ab^ (0.743–0.762)	78.20	0.706 0.647	**0.769** ^**abc**^ **(0.761–0.778)**	0.498	0.715 0.694	0.648^abc^ (0.638–0.659)	0.0758	0.682 0.537	**0.769** ^**abc**^ **(0.761–0.778)**	3.33	0.717 0.694	0.711^abc^ (0.701–0.721)	1.190	0.660 0.657
Diabetes/IFG	0.699^a^ (0.693–0.705)	23.82	0.692 0.606	0.710^a^ (0.704–0.716)	79.25	0.606 0.704	**0.722** ^**abc**^ **(0.716–0.727)**	0.490	0.695 0.633	0.626^abc^ (0.520–0.632)	0.0769	0.561 0.627	**0.722** ^**abc**^ **(0.716–0.727)**	3.18	0.695 0.633	0.676^abc^ (0.670–0.682)	1.180	0.656 0.601
High TG	0.729^ac^ (0.723–0.734)	23.00	0.690 0.655	0.741^ab^ (0.735–0.747)	77.25	0.713 0.647	**0.752** ^**abc**^ **(0.746–0.758)**	0.489	0.725 0.652	0.638^abc^ (0.632–0.644)	0.0770	0.556 0.651	**0.752** ^**abc**^ **(0.746–0.758)**	3.16	0.725 0.652	0.697^ac^ (0.691–0.703)	1.173	0.711 0.585
Low HDL-C	**0.644** ^**a**^ **(0.637–0.650)**	22.21	0.613 0.601	0.643^a^ (0.637–0.649)	75.75	0.599 0.612	0.643^a^ (0.637–0.650)	0.473	0.624 0.589	0.564^abc^ (0.558–0.571)	0.0746	0.640 0.458	0.643^a^ (0.637–0.650)	2.87	0.624 0.589	0.603^abc^ (0.597–0.609)	1.143	0.684 0.465
High LDL-C	0.628^ac^ (0.622–0.634)	22.29	0.643 0.543	0.635^ab^ (0.629–0.641)	76.75	0.608 0.597	**0.645** ^**abc**^ **(0.639–0.652)**	0.474	0.678 0.533	0.577^abc^ (0.570–0.583)	0.0764	0.515 0.599	**0.645** ^**abc**^ **(0.639–0.652)**	2.89	0.678 0.533	0.611^abc^ (0.605–0.617)	1.173	0.594 0.576
Hyperuricemia	0.722^a^ (0.716–0.728)	22.96	0.729 0.607	0.724^a^ (0.718–0.730)	79.25	0.657 0.683	**0.728** ^**a**^ **(0.722–0.733)**	0.502	0.665 0.675	0.609^abc^ (0.603–0.615)	0.0759	0.636 0.532	**0.728** ^**a**^ **(0.722–0.733)**	3.39	0.665 0.675	0.671^abc^ (0.655–0.677)	1.187	0.643 0.615
NAFLD	**0.837** ^**ac**^ **(0.833–0.842)**	23.26	0.806 0.718	0.831^ab^ (0.826–0.836)	78.45	0.795 0.715	0.832^ab^ (0.827–0.836)	0.498	0.782 0.732	0.643^abc^ (0.636–0.649)	0.0763	0.612 0.599	0.832^ab^ (0.827–0.837)	3.33	0.782 0.732	0.738^abc^ (0.732–0.744)	1.182	0.716 0.644
*Men*																		
Hypertension	0.667^ac^ (0.662–0.672)	24.75	0.705 0.542	0.673^ab^ (0.668–0.678)	88.25	0.662 0.584	**0.690** ^**abc**^ **(0.685–0.695)**	0.524	0.665 0.614	0.586^abc^ (0.581–0.591)	0.0790	0.604 0.523	**0.690** ^**abc**^ **(0.685–0.695)**	3.81	0.665 0.614	0.643^abc^ (0.638–0.648)	1.243	0.647 0.562
Diabetes/IFG	0.649^ac^ (0.644–0.654)	24.79	0.668 0.546	0.658^ab^ (0.653–0.663)	89.90	0.594 0.637	**0.667** ^**abc**^ **(0.662–0.672)**	0.526	0.610 0.629	0.580^abc^ (0.575–0.585)	0.0800	0.493 0.624	**0.667** ^**abc**^ **(0.662–0.672)**	3.86	0.610 0.629	0.630^abc^ (0.652–0.635)	1.247	0.597 0.592
High TG	0.686^a^ (0.681–0.691)	24.32	0.720 0.553	0.688^a^ (0.683–0.693)	85.80	0.761 0.515	**0.689** ^**a**^ **(0.684–0.694)**	0.504	0.763 0.516	0.570^abc^ (0.565–0.575)	0.0789	0.584 0.524	**0.689** ^**a**^ **(0.684–0.694)**	3.44	0.763 0.516	0.634^abc^ (0.629–0.639)	1.227	0.688 0.509
Low HDL-C	**0.641** ^**ac**^ **(0.636–0.646)**	24.24	0.687 0.520	0.636^ab^ (0.631–0.641)	85.80	0.712 0.496	0.632^abc^ (0.627–0.637)	0.510	0.662 0.534	0.535^abc^ (0.529–0.540)	0.0775	0.689 0.363	0.632^abc^ (0.627–0.637)	3.55	0.662 0.534	0.584^abc^ (0.579–0.589)	1.233	0.612 0.512
High LDL-C	0.567^a^ (0.562–0.573)	23.60	0.728 0.373	0.565^a^ (0.560–0.570)	84.75	0.715 0.383	**0.569** ^**ac**^ **(0.564–0.574)**	0.494	0.755 0.358	0.524^abc^ (0.519–0.530)	0.0780	0.644 0.396	**0.569** ^**ac**^ **(0.564–0.574)**	3.24	0.755 0.358	0.547^abc^ (0.542–0.552)	1.207	0.743 0.331
Hyperuricemia	**0.649** ^**a**^ **(0.644–0.654)**	25.14	0.638 0.580	0.646^a^ (0.641–0.651)	86.25	0.740 0.472	0.638^abc^ (0.633–0.643)	0.503	0.784 0.414	0.532^abc^ (0.527–0.538)	0.0789	0.566 0.485	0.638^abc^ (0.633–0.643)	3.41	0.781 0.418	0.588^abc^ (0.583–0.593)	1.220	0.728 0.403
NAFLD	**0.770** ^**ac**^ **(0.766–0.775)**	24.87	0.721 0.692	0.763^ab^ (0.759–0.767)	87.75	0.733 0.666	0.757^abc^ (0.752–0.761)	0.513	0.748 0.640	0.572^abc^ (0.566–0.577)	0.0784	0.640 0.471	0.757^abc^ (0.752–0.761)	3.59	0.749 0.638	0.666^abc^ (0.661–0.671)	1.231	0.685 0.557

AUC: area under the curve; ROC: receiver-operating characteristic; BMI: body mass index; WC: waist circumference; WHtR: waist-to-height ratio; ABSI: a body shape index; BRI: body roundness index; CI: conicity index; 95% CI: 95% confidence interval; Sen: sensitivity; Spe: specificity; IFG: impaired fasting glucose; TG: triglycerides; HDL-C: high-density lipoprotein cholesterol; LDL-C: low-density lipoprotein cholesterol; NAFLD: nonalcoholic fatty liver. AUC in the ROC analysis, ^a^ *p* < 0.001. Hanley and McNeil's approach was used to compare the AUCs of the indices. ^b^ *p* < 0.05, compared with the AUC of BMI; ^c^ *p* < 0.05, compared with the AUC of WC. The bold indicates the highest AUC value among the indices.

**Table 5:** AUCs, optimal cut-off points, and sensitivities and specificities of the cut-off points of the anthropometric indices for predicting MetS in the ROC analyses according to age group.

Index	Age group	Women					Men				
		AUC	95% CI	Cut-off	Sen	Spe	AUC	95% CI	Cut-off	Sen	Spe
BMI	Global	0.760^ac^	0.753–0.767	23.03	0.704	0.688	0.719^ac^	0.714–0.724	24.64	0.709	0.613
	High sensitivity			22.27	0.800	0.580			23.91	0.800	0.511
	<45	**0.761** ^**ac**^	0.747–0.775	22.10	0.746	0.650	0.734^a^	0.727–0.742	24.88	0.690	0.657
	45–59	0.699^a^	0.688–0.711	23.52	0.657	0.638	**0.700** ^**a**^	0.692–0.709	24.65	0.711	0.581
	≥60	0.673^a^	0.650–0.696	23.45	0.694	0.568	0.722^a^	0.706–0.738	24.56	0.686	0.650

WC	Global	0.770^ab^	0.763–0.777	77.25	0.726	0.677	0.721^ab^	0.716–0.726	87.25	0.703	0.621
	High sensitivity			75.75	0.809	0.589			84.75	0.826	0.478
	<45	0.740^ab^	0.726–0.755	74.75	0.704	0.655	0.731^a^	0.723–0.738	85.80	0.750	0.587
	45–59	0.701^a^	0.689–0.712	78.75	0.700	0.587	0.695^a^	0.687–0.703	87.25	0.737	0.546
	≥60	0.672^a^	0.649–0.695	80.25	0.751	0.506	**0.723** ^**a**^	0.707–0.740	88.25	0.678	0.645

WHtR	Global	**0.782** ^**abc**^	0.776–0.789	0.490	0.735	0.689	**0.727** ^**abc**^	0.721–0.732	0.510	0.748	0.584
	High sensitivity			0.480	0.800	0.618			0.503	0.800	0.524
	<45	0.751^abc^	0.737–0.765	0.477	0.654	0.716	**0.736** ^**ac**^	0.729–0.744	0.502	0.752	0.601
	45–59	**0.707** ^**ac**^	0.695–0.718	0.498	0.698	0.604	0.698^a^	0.690–0.706	0.524	0.668	0.625
	≥60	**0.676** ^**a**^	0.653–0.699	0.531	0.669	0.592	0.720^a^	0.703–0.736	0.529	0.689	0.638

ABSI	Global	0.648^abc^	0.640–0.657	0.0769	0.565	0.655	0.585^abc^	0.579–0.591	0.0790	0.586	0.540
	High sensitivity			0.0738	0.800	0.379			0.0767	0.800	0.307
	<45	0.569^abc^	0.552–0.585	0.0731	0.712	0.394	0.570^abc^	0.561–0.578	0.0765	0.716	0.386
	45–59	0.581^abc^	0.568–0.593	0.0772	0.533	0.589	0.559^abc^	0.550–0.568	0.0789	0.671	0.426
	≥60	0.571^abc^	0.547–0.596	0.0781	0.732	0.397	0.582^abc^	0.563–0.600	0.0801	0.672	0.466

BRI	Global	**0.782** ^**abc**^	0.776–0.789	3.179	0.735	0.689	**0.727** ^**abc**^	0.721–0.732	3.547	0.748	0.584
	High sensitivity			2.996	0.800	0.618			3.413	0.800	0.524
	<45	0.751^abc^	0.737–0.765	2.931	0.654	0.716	**0.736** ^**ac**^	0.729–0.744	3.388	0.752	0.602
	45–59	**0.707** ^**ac**^	0.695–0.718	3.329	0.699	0.604	0.698^a^	0.690–0.706	3.818	0.668	0.625
	≥60	**0.676** ^**a**^	0.653–0.699	3.963	0.669	0.592	0.720^a^	0.703–0.736	3.929	0.689	0.638

CI	Global	0.716^abc^	0.709–0.724	1.178	0.685	0.635	0.660^abc^	0.655–0.666	1.231	0.688	0.548
	High sensitivity			1.151	0.800	0.494			1.211	0.800	0.416
	<45	0.655^abc^	0.639–0.670	1.144	0.669	0.565	0.661^abc^	0.652–0.669	1.214	0.693	0.544
	45–59	0.640^abc^	0.628–0.652	1.190	0.634	0.571	0.630^abc^	0.621–0.639	1.248	0.649	0.544
	≥60	0.623^abc^	0.600–0.647	1.234	0.680	0.517	0.654^abc^	0.637–0.672	1.281	0.548	0.687

AUC: area under the curve; MetS: metabolic syndrome; ROC: receiver-operating characteristic; 95% CI: 95% confidence interval; Sen: sensitivity; Spe: specificity; BMI: body mass index; WC: waist circumference; WHtR: waist-to-height ratio; ABSI: a body shape index; BRI: body roundness index; CI: conicity index. AUC in the ROC analysis, ^a^ *p* < 0.001. Hanley and McNeil's approach was used to compare the AUCs of the indices. ^b^ *p* < 0.05, compared with the AUC of BMI; ^c^ *p* < 0.05, compared with the AUC of WC. The bold indicates the highest AUC value among the indices.

**Table 6:** AUCs, optimal cut-off points, and sensitivities and specificities of the cut-off points of the anthropometric indices for predicting MetS in the ROC analyses according to BMI category.

BMI category	Index	Women					Men				
		AUC	95% CI	Cut-off	Sen	Spe	AUC	95% CI	Cut-off	Sen	Spe
Underweight (BMI of <18.50 kg/m^2^)	BMI	0.468^a^	0.360–0.576	18.42	0.129	0.934	0.499^a^	0.373–0.626	18.06	0.455	0.628
	WC	0.621^a^	0.518–0.724	65.25	0.581	0.622	0.498^a^	0.366–0.629	73.75	0.318	0.827
	WHtR	0.660^ab^	0.557–0.762	0.422	0.484	0.779	**0.562** ^**a**^	0.447–0.677	0.387	0.909	0.250
	ABSI	**0.676** ^**abc**^	0.580–0.772	0.0775	0.581	0.707	0.548^a^	0.416–0.680	0.0836	0.273	0.893
	BRI	0.660^ab^	0.557–0.762	2.020	0.484	0.779	**0.562** ^**a**^	0.447–0.677	1.484	0.909	0.250
	CI	0.669^ac^	0.571–0.766	1.133	0.645	0.641	0.543^a^	0.414–0.672	1.236	0.273	0.884

Normal weight (BMI of 18.50–23.99 kg/m^2^)	BMI	0.674^ac^	0.663–0.686	21.68	0.690	0.578	0.656^ac^	0.645–0.666	22.43	0.669	0.574
	WC	0.705^ab^	0.694–0.716	74.25	0.678	0.629	0.669^ab^	0.659–0.679	81.75	0.672	0.576
	WHtR	**0.729** ^**abc**^	0.718–0.740	0.477	0.631	0.704	**0.680** ^**abc**^	0.670–0.690	0.477	0.717	0.552
	ABSI	0.656^abc^	0.644–0.668	0.0769	0.561	0.669	0.613^abc^	0.602–0.624	0.0792	0.599	0.574
	BRI	**0.729** ^**abc**^	0.718–0.740	2.939	0.631	0.704	**0.680** ^**abc**^	0.670–0.690	2.937	0.717	0.552
	CI	0.682^ac^	0.671–0.694	1.166	0.644	0.626	0.640^abc^	0.629–0.651	1.217	0.637	0.572

Overweight (BMI of 24–27.99 kg/m^2^)	BMI	0.575^ac^	0.560–0.591	25.19	0.631	0.491	0.575^ac^	0.566–0.583	25.89	0.516	0.597
	WC	0.618^ab^	0.603–0.633	83.75	0.582	0.597	0.598^ab^	0.589–0.606	89.90	0.615	0.526
	WHtR	**0.629** ^**abc**^	0.614–0.644	0.534	0.561	0.639	**0.607** ^**abc**^	0.599–0.616	0.529	0.591	0.567
	ABSI	0.609^abc^	0.594–0.624	0.0772	0.534	0.638	0.581^ac^	0.573–0.590	0.0791	0.576	0.544
	BRI	0.629^abc^	0.614–0.644	4.020	0.561	0.639	**0.607** ^**abc**^	0.599–0.616	3.927	0.592	0.566
	CI	0.617^ab^	0.601–0.632	1.215	0.545	0.636	0.592^abc^	0.583–0.600	1.250	0.561	0.573

Obesity (BMI of ≥28.00 kg/m^2^)	BMI	0.549^ac^	0.515–0.583	32.15	0.154	0.940	0.558^a^	0.542–0.575	29.27	0.552	0.545
	WC	**0.608** ^**ab**^	0.574–0.641	91.75	0.650	0.509	0.572^a^	0.556–0.588	100.25	0.417	0.690
	WHtR	**0.608** ^**ab**^	0.575–0.641	0.590	0.588	0.584	0.577^ab^	0.560–0.593	0.578	0.594	0.524
	ABSI	0.596^ab^	0.563–0.630	0.0773	0.565	0.600	0.553^ac^	0.536–0.569	0.0793	0.505	0.578
	BRI	0.608^ab^	0.575–0.641	5.194	0.588	0.584	0.577^ab^	0.560–0.593	4.940	0.594	0.524
	CI	0.606^ab^	0.572–0.639	1.250	0.569	0.619	0.565^a^	0.549–0.581	1.272	0.574	0.525

AUC: area under the curve; MetS: metabolic syndrome; ROC: receiver-operating characteristic; BMI: body mass index; 95% CI: 95% confidence interval; Sen: sensitivity; Spe: specificity; WC: waist circumference; WHtR: waist-to-height ratio; ABSI: a body shape index; BRI: body roundness index; CI: conicity index. AUC in the ROC analysis, ^a^ *p* < 0.001. Hanley and McNeil's approach was used to compare the AUCs of the indices. ^b^ *p* < 0.05, compared with the AUC of BMI; ^c^ *p* < 0.05, compared with the AUC of WC. The bold indicates the highest AUC value among the indices.

**Table 7:** Logistic regression analyses of the association of the cardiometabolic risk factors and MetS with each anthropometric index according to BMI category, indicated by ORs.

BMI category	Index	Female								Male							
		Hypertension	Diabetes/IFG	High TG	Low HDL-C	High LDL-C	Hyperuricemia	NAFLD	MetS	Hypertension	Diabetes/IFG	High TG	Low HDL-C	High LDL-C	Hyperuricemia	NAFLD	MetS
Underweight (BMI of <18.50 kg/m^2^)	High WC	—	—	—	1.448^b^	—	—	—	—	—	—	—	—	—	—	—	—
	High WHtR	—	—	—	—	—	—	—	—	—	—	—	—	—	—	—	—
	High ABSI	—	—								—					—	—
	High BRI	—	—	—	—	—	—	—	—	—	—	—	—	—	—	—	—
	High CI	—	—	—	—	—	—	—	—	—	—	—	—	—	—	—	—

Normal weight (BMI of 18.50–23.99 kg/m^2^)	High WC	**1.409** ^**a**^	1.443^a^	2.281^a^	1.738^a^	1.210^a^	**1.860** ^**a**^	**3.079** ^**a**^	2.283^a^	1.666^a^	1.563^a^	2.603^a^	1.866^a^	1.466^a^	2.079^a^	2.882^a^	2.506^a^
	High WHtR	1.406^a^	1.566^a^	2.310^a^	1.770^a^	1.187^a^	1.835^a^	1.835^a^	2.398^a^	**1.807** ^**a**^	**1.596** ^**a**^	**2.845** ^**a**^	**1.907** ^**a**^	**1.583** ^**a**^	**2.101** ^**a**^	**3.014** ^**a**^	**2.781** ^**a**^
	High ABSI	—	1.212^b^	1.634^a^	1.333^a^	1.124^a^	1.338^b^	1.645^a^	1.572^a^	1.176^b^	1.181^b^	1.937^a^	1.414^a^	1.237^a^	1.592^a^	1.827^a^	1.695^a^
	High BRI	1.406^a^	**1.569** ^**a**^	**2.318** ^**a**^	**1.773** ^**a**^	**1.191** ^**a**^	1.846^a^	2.980^a^	**2.409** ^**a**^	**1.807** ^**a**^	**1.596** ^**a**^	**2.845** ^**a**^	**1.907** ^**a**^	**1.583** ^**a**^	**2.101** ^**a**^	**3.014** ^**a**^	**2.718** ^**a**^
	High CI	—	1.311^a^	1.894^a^	1.461^a^	1.170^a^	1.548^a^	1.960^a^	1.821^a^	1.273^a^	1.265^a^	2.252^a^	1.582^a^	1.377^a^	1.850^a^	2.183^a^	1.996^a^

Overweight (BMI of 24–27.99 kg/m^2^)	High WC	**1.310** ^**a**^	1.407^a^	1.508^a^	1.315^a^	—	**1.928** ^**a**^	**1.976** ^**a**^	1.544^a^	1.349^a^	1.479^a^	1.584^a^	1.323^a^	1.112^b^	1.508^a^	2.134^a^	1.651^a^
	High WHtR	1.192^c^	**1.548** ^**a**^	**1.554** ^**a**^	**1.336** ^**a**^	**1.192** ^**b**^	1.853^a^	1.795^a^	**1.571** ^**a**^	1.436^a^	1.342^a^	1.661^a^	1.330^a^	1.094^b^	1.465^a^	1.959^a^	1.683^a^
	High ABSI	—	1.340^a^	1.432^a^	1.307^a^	—	1.591^a^	1.548^a^	1.390^a^	1.173^a^	1.207^a^	1.474^a^	1.247^a^	—	1.277^a^	1.496^a^	1.430^a^
	High BRI	1.192^c^	**1.548** ^**a**^	**1.554** ^**a**^	**1.336** ^**a**^	**1.192** ^**b**^	1.853^a^	1.795^a^	**1.571** ^**a**^	**1.444** ^**a**^	1.358^a^	1.671^a^	1.336^a^	1.090c	1.466^a^	1.961^a^	1.698^a^
	High CI	—	1.422^a^	1.418^a^	1.340^a^	1.135^c^	1.683^a^	1.665^a^	1.440^a^	1.235^a^	1.305^a^	1.547^a^	1.301^a^	1.096^b^	1.330^a^	1.698^a^	1.530^a^

Obesity (BMI of ≥28.00 kg/m^2^)	High WC	—	**2.115** ^**a**^	1.379^c^	1.445^c^	—	1.756^b^	**1.787** ^**a**^	**1.663** ^**a**^	**1.432** ^**a**^	**1.530** ^**a**^	1.278^a^	1.167^b^	—	1.231^b^	1.199^b^	1.524^a^
	High WHtR	—	1.875^a^	1.391^b^	—	—	1.981^a^	1.657^a^	1.569^a^	1.314^a^	1.435^a^	**1.343** ^**a**^	—	—	**1.260** ^**a**^	**1.246** ^**b**^	**1.463** ^**a**^
	High ABSI	—	1.325^c^	1.326^c^	1.479^b^	—	—	—	1.443^b^	—	1.238^a^	1.258^a^	—	—	—	1.148^c^	1.198^b^
	High BRI	—	1.898^a^	**1.403** ^**b**^	—	—	**2.031** ^**a**^	1.667^a^	1.577^a^	1.314^a^	1.435^a^	**1.343** ^**a**^	—	—	**1.260** ^**a**^	**1.246** ^**b**^	**1.463** ^**a**^
	High CI	—	1.515^b^	1.527^b^	**1.497** ^**b**^	—	1.495^c^	1.416^c^	1.625^a^	1.158^c^	1.329^a^	1.265^a^	—	—	1.147^c^	1.169^c^	1.308^a^

MetS: metabolic syndrome; BMI: body mass index; OR: odds ratio; IFG: impaired fasting glucose; TG: triglycerides; HDL-C: high-density lipoprotein cholesterol; LDL-C: low-density lipoprotein cholesterol; NAFLD: nonalcoholic fatty liver disease; WC: waist circumference; WHtR: waist-to-height ratio; ABSI: a body shape index; BRI: body roundness index; CI: conicity index. The regression analyses were adjusted for age. ^a^ *p* < 0.001, ^b^ *p* < 0.01, ^c^ *p* < 0.05. The bold indicates the highest value of odds ratio among the indices.

## Data Availability

The data used to support the findings of this study are available from the corresponding author upon request.

## Supplementary Materials

Supplementary Materials Table S1: correlations among anthropometric indices of body composition in nondiabetic subjects.
