# Supplementary material for: Sex- and Age-Specific Optimal Anthropometric Indices as Screening Tools for Metabolic Syndrome in Chinese Adults
Source: Int J Endocrinol. 2018 Sep 17;2018:1067603. doi: 10.1155/2018/1067603 (PMC6166375; doi:10.1155/2018/1067603)
Supplement: Supplementary Materials — Table S1: correlations among anthropometric indices of body composition in nondiabetic subjects. [file 1067603.f1.docx]

| **Table 1**  Correlations among anthropometric indices of body composition in non-diabetic subjects | | | | | | | | |
| --- | --- | --- | --- | --- | --- | --- | --- | --- |
| **Index** | **Weight** | **Height** | **BMI** | **WC** | **WHtR** | **ABSI** | **BRI** | **CI** |
| **Female** |  |  |  |  |  |  |  |  |
| Weight | 1 | 0.424^a^ | **0.874^a^** | 0.786^a^ | 0.638^a^ | - | 0.636^a^ | 0.289^a^ |
| Height | 0.339^a^ | 1 | -0.065^a^ | 0.139^a^ | -0.189^a^ | 0.049^a^ | -0.186^a^ | 0.024^a^ |
| BMI | 0.874^a^ | -0.157^a^ | 1 | 0.739^a^ | **0.807^a^** | -0.025^a^ | **0.805^a^** | 0.307^a^ |
| WC | 0.782^a^ | - | 0.824^a^ | 1 | **0.945^a^** | 0.564^a^ | **0.939^a^** | **0.800^a^** |
| WHtR | 0.646^a^ | -0.295^a^ | 0.833^a^ | 0.956^a^ | 1 | 0.544^a^ | **0.995^a^** | 0.786^a^ |
| ABSI | 0.085^a^ | -0.059^a^ | 0.120^a^ | 0.639^a^ | 0.628^a^ | 1 | 0.534^a^ | **0.943^a^** |
| BRI | 0.646^a^ | -0.292^a^ | 0.832^a^ | 0.952^a^ | 0.996^a^ | 0.619^a^ | 1 | 0.775^a^ |
| CI | 0.353^a^ | -0.105^a^ | 0.425^a^ | 0.844^a^ | 0.837^a^ | 0.949^a^ | 0.828^a^ | 1 |
| **Males** |  |  |  |  |  |  |  |  |
| Weight | 1 | 0.460^a^ | **0.879^a^** | **0.854^a^** | 0.699^a^ | 0.047^a^ | 0.696^a^ | 0.404^a^ |
| Height | 0.462^a^ | 1 | -0.015^b^ | 0.198^a^ | -0.149^a^ | 0.083^a^ | -0.150^a^ | 0.069^a^ |
| BMI | 0.871^a^ | -0.028^a^ | 1 | **0.858^a^** | **0.871^a^** | - | **0.869^a^** | 0.421^a^ |
| WC | 0.826^a^ | 0.144^a^ | 0.853^a^ | 1 | **0.939^a^** | 0.489^a^ | **0.935^a^** | 0.799^a^ |
| WHtR | 0.655^a^ | -0.205^a^ | 0.853^a^ | 0.939^a^ | 1 | 0.466^a^ | **0.997^a^** | 0.783^a^ |
| ABSI | 0.019^a^ | -0.013^c^ | 0.029^a^ | 0.513^a^ | 0.513^a^ | 1 | 0.460^a^ | **0.910^a^** |
| BRI | 0.654^a^ | -0.205^a^ | 0.853^a^ | 0.935^a^ | 0.997^a^ | 0.505^a^ | 1 | 0.775^a^ |
| CI | 0.354^a^ | -0.204^a^ | 0.414^a^ | 0.799^a^ | 0.799^a^ | 0.921^a^ | 0.792^a^ | 1 |
| BMI, body mass index; WC, waist circumference; WHtR, waist-to-height ratio; ABSI, a body shape index; BRI, body roundness index; CI, conicity index.  Left side (below diagonal) shows correlation of the crude value; right side (above diagonal) shows the partial correlation adjusted for age.  Z-scores for theses anthropometric indices were used in the correlation analyses.  ^a^*p* <0.001, ^b^*p*＜0.01, ^c^*p*＜0.05  The bold indicates the strong correlation between two indices. | | | | | | | | |

**Table 1** shows the correlations among the anthropometric indices. After adjustment for age, the partial correlation analyses showed the strong correlations between BMI and weight, WHtR and BMI, WHtR and WC, BRI and BMI, BRI and WC, BRI and WHtR, CI and WC, CI and ABSI in females, as well as BMI and weight, WC and weight, WC and BMI, WHtR and BMI, WHtR and WC, BRI and BMI, BRI and WC, BRI and WHtR, CI and ABSI in males.
